# Effect of Turmeric and Aloe Vera Extract on Shelf-Life of Goat and Buffalo Admixture Milk Paneer during Refrigeration Storage

**DOI:** 10.3390/foods11233870

**Published:** 2022-11-30

**Authors:** Pramila Umaraw, Akhilesh K. Verma, V. P. Singh, Ahmad Fahim

**Affiliations:** 1 Department of Livestock Products Technology, College of Veterinary and Animal Sciences, Sardar Val-labhbhai Patel University of Agriculture and Technology, Meerut 250110, India; 2 LPM, College of Veterinary and Animal Sciences, Sardar Vallabhbhai Patel University of Agriculture and Technology, Meerut 250110, India

**Keywords:** goat paneer, turmeric, aloe vera, antioxidant activity, storage stability

## Abstract

The present study was undertaken to evaluate efficacy of turmeric and aloe vera extract in extending shelf life of goat milk paneer. The paneer was made by admixing goat milk and buffalo milk in the ratio of 60:40 so as to achieve a standard fat 4.5%. The treatment group, T1, was made by adding 5 mL/100 mL (*v*/*v*) of turmeric extract in heated milk before coagulation. Similarly, 5 mL/100 mL (*v*/*v*) aloe vera extract was added to heated milk for group T2 while T3 was prepared by adding both turmeric extract 5 mL/100 mL (*v*/*v*) and aloe vera extract 5 mL/100 mL (*v*/*v*), and the control was made without any additive. The extracts before incorporation were assessed for their antioxidant and antimicrobial potential by analysing total phenolic content, ABTS and DPPH percent inhibition and zone of inhibition. The developed paneer samples were evaluated for physico-chemical, oxidative and microbiological changes, and sensory attributes during storage at refrigeration temperature for ten days. The results revealed that paneer prepared with addition of extracts significantly (*p* < 0.05) suppressed physico-chemical deterioration. Significantly (*p* < 0.05) lower peroxide value, TBARS, FFA and microbial counts were noticed in T3 than T1, T2 and the control. The sensory attributes were also better (*p* < 0.05) maintained in T3 during storage. The results concluded that the combination of turmeric and aloe vera extract significantly improves the shelf life of paneer under refrigeration storage and these might be used as phyto-preservatives in paneer.

## 1. Introduction

The goat is the animal of the future for environmental sustainability. With the scarcity of land and water, as well as the expansion of hot and arid climatic conditions, the goat may emerge as the most sustainable source of meat and milk in the future, as goats thrive well in hot and arid climate, feeding off bushes which are otherwise inedible for humans and other livestock. Globally, goat milk and milk products have gained immense attention from researchers and consumers owing to its high therapeutic potential. The goat milk market in India is expected to increase at a 3.8% CAGR and might reach USD 11.4 billion in market size in 2026 [1], with its high nutritional profile and therapeutic value being key drivers. Because of its low yield and distinctive flavour, goat milk is not widely used in the preparation of common milk products such as ghee, khoa, sweets, or paneer, limiting its added value. Although an admixture of goat milk with cow milk and buffalo milk has been used for the preparation of various indigenous products such as cottage cheese, paneer, yoghurt, burfi, Kalandhi, etc. The major limiting factor in most milk products is shelf life or storage stability. Milk products are rich in fats which tend to oxidise and lead to spoilage. Utilisation of phyto-preservatives has recently gained much attention and they have been used for the preservation of meat, bread and milk [2,3,4,5].

Turmeric has shown to possess health-promoting properties due to the presence of curcuminoids. Similarly, aloe vera has also been reported to possess promising antimicrobial and antioxidant activities. Nevertheless, utilisation of both as aqueous extracts in the preservation of paneer has not yet been explored. The aim of our study was to develop goat milk paneer with desired characteristics and its preservation under refrigeration. In preliminary trials, goat and buffalo milk were chosen for development of paneer, because of their good yield and desirable characteristic (data not shown). The present study was undertaken with the objective of evaluating the efficiency of turmeric extract, aloe vera extract and a combination of both in extending the shelf life of goat and buffalo admixture milk paneer. To our knowledge no such study has been done to indicate the effects of these extracts on quality and storage characteristics of goat paneer.

## 2. Materials and Methods

Goat and buffalo milk for preparation of paneer were obtained from the Livestock Research Centre, College of Veterinary and Animal Sciences (COVAS), SVPUAT, Meerut. Turmeric powder (MDH^®^) was purchased from a local market in Meerut, and aloe vera powder was prepared in the laboratory, Livestock Products Technology (LPT), COVAS. All chemicals and media were obtained from standard firms (Himedia, SRL and CDH etc.). Low density polyethylene (LDPE 200 gauge) for packaging of samples was also purchased from the local market in Meerut.

### 2.1. Preparation of Extract and Paneer Samples

Aloe vera whole leaves were cleaned and dried in a hot air oven at 55 ± 5 °C until the weight of the dried leaves became constant. Afterwards, the dried leaves were pulverised in a food mixer and strained through a stainless-steel sieve. Extraction began by dissolving 20 g turmeric and aloe vera powder separately in a pre-weighed 500 mL flask containing 200 mL distilled water at room temperature. Extraction was then carried out by using a magnetic stirrer. Upon completion, the supernatant was collected from the solution by centrifuging it at 1400× *g* for 5 min. To remove the solvent, the recovered extract was concentrated in a rotary vacuum evaporator. The concentration of collected extract was determined by drying it in a hot air oven at 100 ± 2 °C for 15–16 h and the final concentration of the extract was adjusted to 120 mg/mL for each sample with the addition of sterile distil water. The extracts were then stored in amber coloured bottles at refrigerated temperature till further use.

Paneer was prepared by adopting the methodology described by [6] with slight modification. Paneer was made by admixing goat milk and buffalo milk in a ratio of 60:40 (Fat 4.5%; SNF 9.0%). The admixture milk was heated to 95 °C for 5 min then it was cooled to 85 °C for coagulation. Coagulation was carried out by the addition of 1% citric acid with mild agitation in order to mix coagulant throughout. Clear whey separation was followed by the straining of coagulum with a muslin cloth. The coagulum was then placed in a rectangular hoop and was pressed for 15 min. The pressed block was then immediately kept in chilled water for 2 h. The paneer block was kept on a tray to remove excess water and was then packed in LDPE for refrigeration storage. Treatments were prepared by adding 5 mL of turmeric extract/100 mL of milk (a concentration of turmeric extract 120 mg/mL) to T1 and adding 5 mL of aloe vera extract/100 mL of milk (a concentration of aloe vera extract 120 mg/mL) to T2, and adding both 5 mL turmeric and 5 mL aloe vera extract to T3 (using aforementioned concentrations for turmeric and aloe vera) in milk at 85 °C before coagulation. After preparation, the paneer samples were stored at refrigeration temperature for 10 days. Evaluation was done on every second day till the end of the storage period.

### 2.2. Analytical Methods

#### 2.2.1. Antioxidant Parameters

##### Sample Preparation for Antioxidant Analysis

Five grams of paneer sample was blended with 20 mL of ethanol and methanol (1:1) for two to three minutes. The solution obtained was filtered through Whatman filter paper (No. 42) in a conical flask. This filtrate or extract was then used for further evaluation of antioxidant parameters.

##### Total Phenolics

The Folin–Ciocalteu reagent method described by Zhang et al. [7] was used to estimate the total phenolic content (TPC) of samples with gallic acid as the standard. The TPC of the sample was expressed as µg of the gallic acid equivalent (GAE) per gram of sample.

##### 2,2′-Azino-Bis (3-Ethylbenzthiazoline-6-Sulphonic Acid) (ABTS^+^) Radical Scavenging Activity

The spectrophotometric method described by Verma et al. [8] was used for estimating of percent inhibition of ABTS^+^ radical.

##### 2,2′-Diphenyl-1-Picrylhydrazyl (DPPH) Radical Scavenging Activity

The methodology described by Verma et al. [9] was adopted for estimating DPPH radical scavenging activity of the samples. For preparation of a sample, 3.9 mL of 250 µM DPPH reagent was taken in a test tube. To this, 1 mL of Tris-HCl buffer (0.1 M, pH 7.4) and 0.1 mL of sample extract was added and mixed thoroughly. The absorbency of samples was then measured by a UV-VIS spectrophotometer in time t = 0 min (*t*_0_) at 517 nm. Samples were then placed in the dark for 20 min and absorbency was noted, i.e., time t = 20 min (*t*_20_). The control was prepared in a similar manner with ethanol and methanol (1:1) except with no sample. DPPH free radical scavenging activity was estimated as follows:

Scavenging activity (% inhibition) = 100 − (*At*_20_*/At*_0_) × 100 (1)

### 2.3. Physio-Chemical Attributes

The pH of the paneer samples was measured by digital meter. Titratable acidity (TA) expressed as Lactic acid (%) was estimated following the procedure described by [10]; BIS under IS: 10,484 for paneer. A water activity meter was utilised for assessing the water activity of samples.

### 2.4. Oxidative Changes

#### 2.4.1. Tyrosine Value

The proteolysis of the paneer samples during storage was measured calorimetrically as tyrosine value. The sample was prepared by mixing 5 g of sample with 10 mL distilled water and 10 mL of trichloroacetic acid (TCA, 0.72 N). It was incubated at room temperature for 10 min and then filtered through Whatman filter paper No. 42. Separately, sodium carbonate/alkali reagent was prepared by adding anhydrous sodium carbonite (75 g of Na_2_CO_2_) and sodium tetra phosphate (10 g) in double distilled water so as to make a final volume of 500 mL. Five millilitre of filtrate previously prepared was mixed with 2 mL distilled water and 10 mL of sodium carbonate/alkali reagent followed by 2 mL of diluted Folin’s phenol reagent (1-part Folin’s phenol reagent: 2-part distilled water). It was mixed well and incubated at room temperature for ten minutes. Intensity of colour developed was measured in the spectrophotometer at 650 nm. The standard curve for the tyrosine value was prepared with TCA filtrate of concentrations 0 μg/5 mL, 10 μg/5 mL, 20 μg/5 mL, etc. up to 100 μg/5 mL filtrate.

#### 2.4.2. Peroxide Value

Changes in the peroxide value of samples during storage were estimated by a method described by [11] with minor modifications.

#### 2.4.3. TBARS

A distillation method described by [12] was used to assess the TBARS value of paneer samples. Its values were presented in terms of mg malonaldehyde/kg of sample.

#### 2.4.4. Free Fatty Acids

Koniecko’s [11] methodology was adopted for evaluating the FFA content of stored paneer samples. The FFA content was estimated by the following formula.

(2) $$\mathrm{Free}\;\mathrm{fatty}\;\mathrm{acid}\;(\mathrm{FFA})\;\%=\frac{0.1\times \mathrm{mL}\;0.1\;N\;\mathrm{alcoholic}\;\mathrm{KOH}\;\times 0.282}{\mathrm{Sample}\;\mathrm{weight}\;\left(g\right)}\times 100$$

### 2.5. Microbiological Evaluation

The antimicrobial potential of turmeric and aloe vera extract was assessed against selected food borne microbes *Bacillus cereus* (MTCC-7190), *Staphylococcus aureus* (MTCC-7443), *Escherichia coli* (MTCC-2921) and *Candida albicans* (MTCC-227) for antimicrobial activities. About 150 μL of each extract was dispensed into a well of 10 mm diameter prepared with sterile cork borer on a petri plate with solidified media. The petri plate was previously seeded with 0.1 mL of inoculums of the aforementioned tested microbes in the range of 10^5^–10^6^ CFU/mL. These petri plates were incubated at 37 °C for 24 h for bacterial cultures on nutrient agar and 25 °C for 5–7 days for *Candida albicans* on potato dextrose agar. Diameters of the zone of inhibition adjacent to the wells were recorded with vernier calipers.

Methods described by [13] were used for assessing the microbiological quality of stored paneer samples. Standard plate count, psychrophile, coliform, and yeast and mould counts were estimated. Total quantities of microbes were estimated by multiplication of the total number of counted colonies with the reciprocal of dilution factor and presented as the colony forming unit per gram (cfu/g).

### 2.6. Sensory Evaluation

A seven-member experienced panel of judges, including teachers and postgraduate students of the College of Veterinary and Animal Sciences, Sardar Vallabhbhai Patel University of Agriculture and Technology, Meerut, examined samples for sensory parameters, such as colour and appearance, flavour, texture, and overall acceptability using a 9-point descriptive scale, where 9 = excellent, 5 = neither like/neither dislike and 1 = extremely poor. Test samples (about 1 cm × 2 cm pieces) were offered to sensory panellists after allotting suitable codes along with potable drinking water. Evaluation was carried out every second day for 10 days of refrigeration storage.

### 2.7. Statistical Analysis

The experiment was repeated thrice, while all sampling evaluations, except for sensory evaluation, were taken in duplicate; thus, six observations (*n* = 2 × 3 = 6) were taken for each sample. For sensory evaluation, seven panellists evaluated the sample for each experiment, for a total of twenty-one observations (*n* = 7 × 3 = 21). SPSS 22.0 (SPSS Inc., Chicago, IL, USA) software was used for analysing the data for ANOVA while Duncan’s multiple range tests and homogeneity tests were used to check the significant difference between the means at 5% level (*p* < 0.05).

## 3. Results

### 3.1. Antioxidant Parameters

#### 3.1.1. Total Phenolics

**Figure 1:**
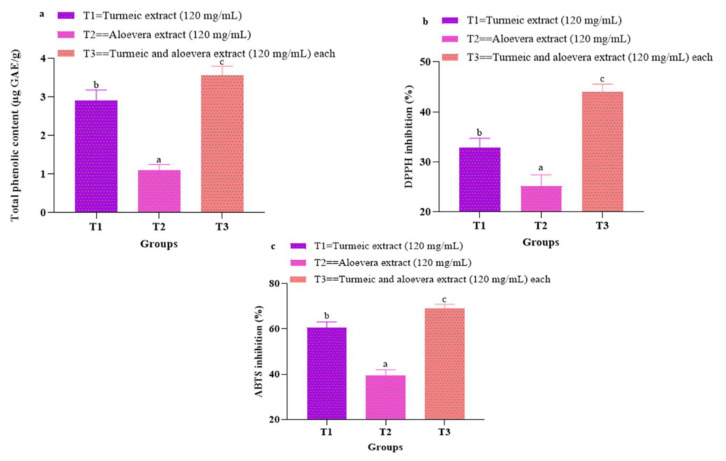
(**a**–**c**) Total phenolic content, DPPH and ABTS (%) inhibition of turmeric, aloe vera and combination of both extracts. Means bearing different superscripts with small letters a, b, c along a row differ significantly at (*p* < 0.05). T1: turmeric extract (120 mg/mL); T2: aloe vera extract (120 mg/mL); T3: turmeric (120 mg/mL) and aloe vera (120 mg/mL) extracts.

Result (Figure 1a) indicates that, of the two extracts prepared, turmeric extract had significantly (*p* < 0.05) higher total phenolic content than aloe vera extract. This difference might be attributed to differences in phytochemical ingredients in plant extract. Aloe vera is a succulent xerophyte of which 99–99.5% is water while the remaining 1–0.5 % constitutes the solid matter rich in phyto-active compounds; by contrast, turmeric is obtained from matured rhizome of turmeric plant which has a higher portion of functional metabolites. Among all extracts, T3, consisting of turmeric and aloe vera, evinced the highest total phenolic content, indicating the additive effect of both the extracts on the phytochemical composition. Several studies [14,15] on incorporation of essential oils in milk and meat products have reported concentration dependent increase in TPC.

#### 3.1.2. 2,2′-Diphenyl-1-Picrylhydrazyl (DPPH) Percent Inhibition

The DPPH percent inhibition of all extracts used evinced significant (*p* < 0.05) variations. The combination of extracts in T3 evinced the highest DPPH percent inhibition followed by T1 and T2 (Figure 1b). The higher activity of T3 might be attributed to a higher level of phytochemicals. Prasad et al. [5] observed that the DPPH free radical scavenging activity of turmeric essential oil was higher than that of ginger and clove essential oil but the combination of all evinced the highest DPPH activity which was attributed to an enhanced level of phytochemicals. Among T1 and T2, T1 evinced significantly (*p* < 0.05) higher DPPH free radical inhibition than T2, which might be attributed to the phytochemicals like curcuminoids present in turmeric extract. Curcuminoids are one of the major phyto-chemicals present in turmeric which has potent antioxidant potential and is also heat stable. In a study, [16] reported that the hydrogen peroxide scavenging activity of curcumin was higher than Trolox, BHT and BHA.

#### 3.1.3. 2,2′-Azino-Bis (3-Ethylbenzthiazoline-6-Sulphonic Acid) (ABTS^+^) Percent Inhibition

The ABTS^+^ radical scavenging activity of turmeric extract (T1), aloe vera extract (T2) and its combination (T3) were significantly (*p* < 0.05) different (Figure 1c). The combination of turmeric and aloe vera (T3) exhibited the highest (*p* < 0.05) percent inhibition, which might be attributed to the additive effect of both extracts. Amongst T1 and T2, the turmeric extract resulted in significantly (*p* < 0.05) better ABTS percent inhibition than T2. Antioxidant activities of plant extracts are highly influenced by its phenolic constituents. The hydroxyl moieties in phenolic compounds transfer hydrogen atoms and scavenge free radicals, preventing oxidation. In a comparative study on antioxidant potential of synthetic and natural polymer additives, [17] reported that the O-H group showed the highest antioxidant activity. It might be postulated that the higher phenolic content of turmeric (T1) was thus more efficient in inhibiting ABTS radicals than that of aloe vera (T2).

### 3.2. Antimicrobial Potential against Selected Bacteria

The antimicrobial potential of three extracts containing turmeric (T1), aloe vera (T2) and a combination of both (T3) was assessed against *B. cereus*, *S. aureus*, *E. coli* and *C. albicans* by agar diffusion assay (Table 1).

All three extracts inhibited growth of *B. cereus* and *C. albicans,* but T2 did not inhibit growth of *S. aureus* and *E. coli*. The zones of inhibition of *S. aureus* and *E. coli* by T1 and T3 were comparable (*p* > 0.05), indicating that the antibacterial effect was primarily due to turmeric. Studies on ethanolic extract of turmeric have also reported significant (*p* < 0.05) inhibition against *S. aureus*, *B. cereus* and *E. faecium,* with zones ranging from 8.88 mm to 17.03 mm [18]. In the present study, aloe vera extract evinced significant (*p* < 0.05) antibacterial activity against only *B. cereus*, with the zone of inhibition ranging from 10.17 mm against *B. cereus* to 12.83 against *C. albicans*. In comparison to turmeric, aloe vera extract showed a slightly larger zone of inhibition against *C. albicans*. Das et al. [19] identified the 15kDa protein from aloe vera leaf gel, which exhibited a potent antimycotic effect against *C. paraprilosis*, *C. krusei* and *C. albicans*.

A combination of extracts, or a cocktail, shows better functional activities than a single extract, due to enhanced and increased levels of phytochemicals, synergistic and/or an additive effect of these. In the present study, it was evident that the combination of turmeric and aloe vera extract exhibited a significantly (*p* < 0.05) larger zone of inhibition against *B. cereus* and *S. aureus* than did single extracts, which might be attributed to the synergistic or additive effects of functional components. The zones of inhibition against *E. coli* and *C. albicans* were comparable to the largest zone shown by either T1 or T2, indicating a non-significant (*p* > 0.05) effect of the combination against these.

### 3.3. Physico-Chemical Attributes

#### 3.3.1. pH

The pH of admixture milk paneer was in the range of 5.72, which was similar to that obtained by Mishra et al. [20] in goat milk paneer prepared with the addition of whey protein concentrates (Table 2). During storage, values did not show significant (*p* > 0.05) differences except for the last day of storage where the control evinced significantly (*p* < 0.05) lowest values. A similar insignificant effect of incorporation of lemon grass extract (by adding it in as a crushed form before boiling) was observed by [21]. It was reported that incorporation of lemon grass at 2%, 4% and 6% did not significantly affect pH values of paneer. During storage, all samples evinced significant (*p* < 0.05) decreases in pH values. The decreases were attributed to the lactic acid production by microorganisms.

#### 3.3.2. Titratable Acidity (TA)

The effect of incorporation of turmeric or aloe vera was insignificant (*p* > 0.05) on day zero (Table 2). Similarly, Buch et al. [22] observed no significant difference in TA of paneer samples prepared with incorporation of turmeric at 2%, 4% and 6% which was added before heating. In another study on heat coagulated milk product “*burfi*”, [5] also evinced no change in acidity by incorporation of turmeric essential oil at the rate of 100 parts per million. However, from the second day, significant (*p* < 0.05) difference in TA was observed between the control and T3. The higher titratable acidity of T3 might be attributed to the additive effect of turmeric and aloe vera extracts. The TA values of all samples increased significantly (*p* < 0.05) by the end of the study. A similar increase in TA during storage was also observed by [23] in “*Kalandhi*”.

#### 3.3.3. Water Activity

Water activity of paneer was not affected by incorporation of turmeric or aloe vera (Table 2). Similarly, Prasad et al. [5] also observed no changes in the a_w_ of burfi made with turmeric essential oil. During storage, the a_w_ of paneer increased in treatment, which was significant (*p* < 0.05) from the second day of storage and remained so till the end of the study. During storage, the a_w_ of all samples decreased significantly (*p* < 0.05), which might be attributed to the loss of moisture during storage. A similar decrease in the a_w_ was also observed by Olivo et al. [24] in ripened cheese wrapped in turmeric incorporated alginate coatings.

### 3.4. Oxidative Changes

#### 3.4.1. Tyrosine Values

**Figure 2:**
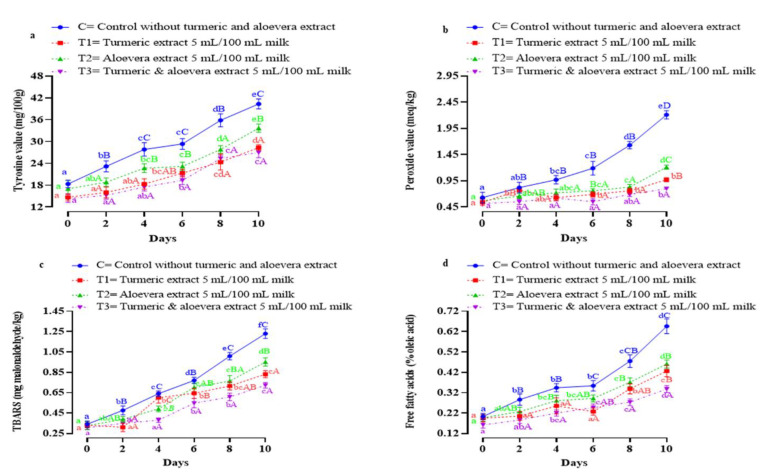
(**a**–**d**) Effect of turmeric and aloe vera extracts on tyrosine value and lipid oxidation attributes of goat and buffalo paneer during refrigerated aerobic storage. Means bearing different superscripts with small letters a, b, c, d and subscripts with capital letters A, B, C, D along a row and column, respectively, differ significantly at (*p* < 0.05). C: Control paneer without turmeric and aloe vera extracts, T1: Paneer with turmeric extract (5 mL/100 mL) *v*/*v* of milk; T2: Paneer with aloe vera extract (5 mL/100 mL) *v*/*v* of milk; T3: paneer with turmeric extract (5 mL/100 mL) *v*/*v* of milk and aloe vera extract (5 mL/100 mL) *v*/*v* of milk.

The tyrosine values in all samples were comparable on the initial day of study but in storage, tyrosine content of all samples increased starting from the second day of analysis (Figure 2a). An increase was significant (*p* < 0.05) in the control and insignificant (*p* > 0.05) in treatments, which might be attributed to the additives turmeric (T1), aloe vera (T2) and the combination of the two (T3). With commencement of storage, the tyrosine values of all samples increased significantly (*p* < 0.05), but the increase was most constrained in T3 and T1. The highest value on the tenth day (27.00 ± 1.45 and 28.32 ± 0.76) of T3 were comparable to the fourth day’s value (27.83 ± 1.85) of the control, indicating that turmeric (T1) and the turmeric and aloe vera combination (T3) were significantly (*p* < 0.05) more efficient in controlling the proteolytic degradation of paneer samples. Gradual proteolytic degradation might be either due to microbial action or enzymatic action. Phytochemicals in turmeric as well as in aloe vera have shown antimicrobial and antioxidant activity. The authors de Carvalho et al. [2] observed that turmeric extract restricted lipid oxidation and free radical generation in lamb sausages. Kebede et al. [25] reported curlone, ar-turmerone and α-Turmerone as major constituents of turmeric essential oils that showed antioxidant and antimicrobial activities.

#### 3.4.2. Peroxide Values

All paneer samples evinced low and comparable peroxide values on the initial day but with storage, values increased at different rates in all samples (Figure 2b). An exponential increase was observed in the control while treatments evinced significantly (*p* < 0.05) slower increases. Turmeric contains curcumin, which is a phenolic compound with potential peroxyl radical scavenging activity. Similar to our findings, Buch et al. [22] observed a slower increase in peroxide value of paneer incorporated with turmeric. Antioxidants like Vitamin E (a-tocopherol), Vitamin C (ascorbic acid) and carotenoids are present in aloe vera [26], which might be ascribed for restricted increase in PV for T2. Miranda et al. [27] also attributed the antioxidant potential of aloe vera to a-tocopherol and ascorbic acid content. However, incorporation of either turmeric or aloe vera constrained an increase in peroxide value, and it was the combination of both that was most effective in controlling lipid oxidation. Among T1 and T2, T1 was more potent in controlling the increase of peroxide value in paneer. The higher efficiency of turmeric than aloe vera might be because of the higher content of curcumin in turmeric than the levels of vitamin C & E in aloe vera. Antioxidants such as phenolic compounds, vitamins C, Vitamin E and flavonoids together show synergistic effects and better antioxidant potentials than individually [28]. A similar effect was also observed in the present study, where the T3 sample containing both turmeric and aloe vera exhibited a slower increase in peroxide value throughout the study, with values being quite low by the end of the ten days.

#### 3.4.3. Thiobarbituric Acid Reacting Substances (TBARS)

The TBARS values of all samples were similar on the initial day of study (Figure 2c). During storage, a significant (*p* < 0.05) increase began in the TBARS value in the control from the second day, while those values in treatments were comparable to their initial day values. The lower values in treatments than in the control indicates that incorporation of turmeric, aloe vera and its combination affected the oxidative process. During the entire study, the highest (*p* < 0.05) TBARAS values were recorded in the control. Constrained TBARS value generation in the treatments might be attributed to their higher phenolic contents than that of the control. Similar lower TBARS values were also observed by Britto et al. [29] in dairy product with plantain syrup and turmeric powder. By the end of the study, the lowest TBARS value was observed in T3 followed by T1 > T2. It can be postulated from this result that turmeric is more efficient in controlling secondary oxidation than aloe vera.

#### 3.4.4. Free Fatty Acid (FFA)

Paneer samples evinced low and comparable free fatty acid content on the initial day (Figure 2d). The storage period significantly (*p* < 0.05) affected FFA content of all samples, which increased from the initial day to the tenth day. The control recorded the highest FFA content, while the lowest was observed in T3 by the end of study. On the sixth day, the free fatty acid content of T2 (aloe vera added) and T3 (turmeric and aloe vera) were comparable, while that of T1 was lowest. These results varied from the results of PV and TBARS, where the combination of extracts evinced highest antioxidant activity.

### 3.5. Microbiological Assessment

The standard plate count of all samples increased significantly (*p* < 0.05) during storage (Table 3). The highest SPC count was observed in the control followed by T2 (aloe vera extract) and T1 (turmeric extract). The SPC value of the control and T2 differed non-significantly (*p* > 0.05). The lower SPC count in T2 than in the control might be attributed to the presence of antimicrobial compounds such as anthraquinones, which inhibit bacterial protein synthesis. While, the lower SPC count in T1 might be attributed to potent antimicrobial compounds such as curcumin, curcuminoids, etc. These compounds have also shown potential invitro antimicrobial action against *E. coli* [24]. The lower SPC in T3 might be attributed to the combination of turmeric and aloe vera extracts. The T3 sample with both turmeric and aloe vera extracts exhibited the lowest SPC by the end of storage. This might be due to interactive and synergistic effects of turmeric and aloe vera components.

The psychrophilic count of all paneer samples was low and insignificantly (*p* > 0.05) different from each other for the initial two days of study (Table 3). However, on the fourth day, a significantly (*p* < 0.05) higher count was observed in the control than in the treatments. Among treatments, the psychrophilic count was lowest in T3, which was below log 2 cfu, followed by T1. In comparison to the second day, an increase in psychrophilic counts was observed in all except T3, indicating that T3 evinced restricted psychrophile growth as values were non-significantly (*p* > 0.05) different from that of first day. This might be due to an additive effect of phytochemicals such as phenols, glycosides, anthraquinones, saponins, flavonoids, tannins, terpenoids, etc. found in turmeric and aloe vera possessing antimicrobial properties. During storage, the psychrophile count in all samples increased significantly (*p* < 0.05). The highest count was observed in the control followed by T2 and T1 while T3 observed the slowest psychrophile growth, which might be attributed to curcuminoids such as curcumin, demethoxycurcumin and bisdemethoxycurcumin in turmeric and anthraquinones in aloe vera, which due to its structural similarity to tetracycline, blocks protein synthesis of bacteria.

Coliforms were absent in all samples on the initial day of preparation (Table 3). However, with the commencement of storage, coliforms appeared in the control sample from the sixth day and increased significantly (*p* < 0.05) by the tenth day. Similarly, T1 and T2 recorded coliform counts from the eighth day. Concordant results were reported by Eresam et al. [30] in paneer samples incorporated with cardamon powder, where coliforms appeared from the seventh day of the study and increased significantly (*p* < 0.05) by the 14th day of contemplation. Coliform counts in T3 appeared only on the last day of analysis and the value was quite low. Both turmeric and aloe vera extracts have evinced antimicrobial activity against both gram positive and gram-negative bacteria. Turmeric essential oil contains bioactive volatile components, such as mono and sesquiterpenoids, and non-volatile components, such as curcumin, dimethoxy curcumin and bisdemethoxy curcumin [31], while aloe vera contains antimicrobial compounds, such as pyrocatechol and anthraquinones [26]. Tyagi et al. [32] reported membrane leakage and damage of gram-negative bacteria on exposure to curcumin, one of the major constituents of turmeric essential oil.

Yeast and mould counts appeared in the control and T2 from the sixth day, which increased significantly (*p* < 0.05) by the end of the study (Table 3). The effect of aloe vera extract on yeast and mould count was not significant (*p* > 0.05), as counts in the control and T2 were similar. By contrast, turmeric extract exhibited potent effect in controlling yeast and mould growth, as no growth as was observed in T1 and T3 till six days of analysis. This might be attributed to the presence of many phytochemicals such as α-pinene, alpha-phellandrene, myrcene, ar-turmerone, turmerone, α-curcumene, zingiberene, turmerones, curcuminiods, geraniol, geraniol acetate, linalol and beta-sesquiphellandrene [33]. These components evince both fungistatic and fungicidal activity by disrupting the membrane function in fungi as well as inhibiting protein and nuclear material synthesis. Similarly, lower yeast and mould count was observed in paneer added with cardamom than in the control [30].

### 3.6. Sensory Evaluation

The colour scores of all samples except T1 received values 8 and above while that of T1 received values 7 and above but statistically values were insignificantly (*p* > 0.05) different (Table 4). Hasneen et al. [4] observed lower appearance scores for yoghurt incorporated with herbal extracts. During storage, colour and appearance scores of all samples decreased significantly (*p* < 0.05), which was more pronounced in the control and T2. By contrast, T1 and T3 recorded significantly (*p* < 0.05) better scores on the tenth day than the control and T2, which might be attributed to phytochemicals present in turmeric and aloe vera. The flavour scores of all samples were similar (*p* > 0.05) on the initial day of the study, although the control received the highest scores. All samples showed insignificant differences (*p* > 0.05) in flavour till the sixth day of storage; thereafter, treatments scored significantly (*p* < 0.05) higher scores than the control, which might be correlated with microbiological and oxidative changes results where turmeric, aloe vera extract and their combination significantly (*p* < 0.05) constrained oxidative and microbiological changes. Flavour scores of all samples decreased significantly (*p* < 0.05) throughout storage. Similarly, [34] observed decreasing flavour scores of fresh cottage cheese added with ginger, clove and thyme essential oils at 0.05% and 0.01% separately during four weeks of storage study. The changes in texture scores were evident from the second day, where T3 received significantly (*p* < 0.05) higher scores than the control, while scores of T2 were comparable to T3. Incorporation of turmeric or aloe vera and its combination maintained textural quality better than the control during storage. Similarly, [34] also observed improved textural quality of cottage cheese added with various essential oils (ginger essential oil, thyme oil, clove oil). On comparing the effects of turmeric and aloe vera extracts, it is evident that aloe vera extract containing paneer samples received better texture scores than turmeric, which might be partially influenced by the characteristic pungency of turmeric. El-Sayed and El-Sayed [35] observed that panellists gave higher scores to fresh cheese added with aloe vera pulp than the control; values increased with increasing levels of incorporation. During four weeks of storage, the texture of aloe vera added cheese was better maintained than that of the control. Paneer made with admixture of goat and buffalo milk had good acceptability. All samples secured high ratings than 8, which did not differ significantly (*p* > 0.05). Although during storage, acceptability of products decreased in scores; among groups, scores remained similar till the fourth day of the study. The effect of extract incorporation was more evident during storage, as turmeric, aloe vera and their combination in T1, T2 and T3, respectively, recorded higher/better overall acceptability, of which the combination T3 recorded scores above 7 till the eighth day of storage. Extracts are rich in phytochemicals which restrict, inhibit and/or slow the rate of deterioration caused by oxidative or microbiological changes. The results of lipid oxidation and microbiological changes also reinforce the results of overall acceptability. Good acceptability of paneer or fresh cottage cheese incorporated with essential oils has been observed by [30].

## 4. Conclusions

Turmeric and aloe vera extract evinced good antioxidative and antimicrobial activities. Among extracts, the combination of the two exhibited the best results. On comparing turmeric and aloe vera extracts, it was evident that the antioxidant potential of turmeric was higher than that of aloe vera. Although the antioxidant potential of combination was superior to extracts used individually, indicating significant synergistic and/or additive effects. It was observed that the aloe vera extract evinced the best antimycotic effect. Contrary to the antioxidant potential, the antimicrobial potential of the combination of extracts was not much improved. Goat and buffalo admixture milk paneer with added phyto-preservative extracts evinced good storage stability till the tenth day at refrigeration. During storage, samples containing the combination of turmeric and aloe vera extracts (T3) observed better physicochemical characteristics, the lowest oxidative changes, restricted microbial proliferation and superior acceptability than T1, T2 and the control. Thus, it can be concluded that the combination of turmeric and aloe vera extracts can be used for extending shelf life of paneer under refrigeration storage.

## Figures and Tables

**Table 1:** Zone of inhibition (mm) of turmeric, aloe vera and a combination of both extract against food borne pathogens.

Microbes	T1	T2	T3
*B. cereus*	$11.50\pm 0.43^{a}$	$10.17\pm 0.47^{a}$	$14.50\pm 0.42^{b}$
*S. aureus*	13.83±0.47	ND	12.50±0.43
*E. coli*	10.00±0.37	ND	10.67±0.49
*C. albicans*	$11.50\pm 0.42^{a}$	$12.83\pm 0.31^{b}$	$13.67\pm 0.42^{b}$

Means bearing different superscripts with small letters a, b along a row differ significantly at *p* ≤ 0.05. T1: turmeric extract (120 mg/mL); T2: aloe vera extract (120 mg/mL); T3: turmeric (120 mg/mL) and aloe vera (120 mg/mL) extracts.

**Table 2:** Effect of turmeric and aloe vera extract on physico-chemical attributes of goat and buffalo paneer during refrigerated aerobic storage.

Groups	0 Day	2 Days	4 Days	6 Days	8 Days	10 Days	Group Means	*p*-Value
**pH**								
C	$5.72\pm 0.02^{c}$	$5.64\pm 0.01^{b}$	$5.62\pm 0.01^{b}$	$5.60\pm 0.03^{b}$	$5.53\pm 0.02^{a}$	$5.49\pm 0.02_{A}^{a}$	5.60±0.01	0.000
T1	$5.69\pm 0.01^{d}$	$5.67\pm 0.01^{cd}$	$5.65\pm 0.01^{cd}$	$5.63\pm 0.01^{bc}$	$5.60\pm 0.02^{ab}$	$5.58\pm 0.01_{B}^{a}$	5.64±0.008	0.000
T2	$5.70\pm 0.01^{d}$	$5.65\pm 0.02^{cd}$	$5.63\pm 0.01^{bc}$	$5.62\pm 0.02^{bc}$	$5.59\pm 0.01^{ab}$	$5.55\pm 0.02_{B}^{a}$	5.63±0.01	0.000
T3	$5.71\pm 0.02^{d}$	$5.67\pm 0.02^{cd}$	$5.65\pm 0.02^{cd}$	$5.63\pm 0.02^{bc}$	$5.60\pm 0.02^{ab}$	$5.56\pm 0.01_{B}^{a}$	5.64±0.01	0.00
Storage mean	$5.71\pm 0.007^{c}$	$5.66\pm 0.008^{b}$	$5.64\pm 0.007^{b}$	$5.62\pm 0.008^{a}$	$5.58\pm 0.01^{a}$	$5.55\pm 0.01^{a}$	−	0.00
*p*-value	0.391	0.442	0.185	0.460	0.104	0.06	0.059	−
**Titratable acidity (% lactic acid)**								
C	$0.383\pm 0.007^{a}$	$0.397\pm 0.008_{A}^{a}$	$0.428\pm 0.008^{b}$	$0.471\pm 0.011_{A}^{c}$	$0.499\pm 0.010_{A}^{d}$	$0.526\pm 0.012_{A}^{d}$.	$0.451\pm 0.01_{A}$	0.000
T1	$0.393\pm 0.006^{a}$	$0.417\pm 0.007_{AB}^{ab}$	$0.441\pm 0.008^{b}$	$0.469\pm 0.012_{A}^{c}$	$0.519\pm 0.012_{AB}^{d}$	0.549$\pm 0.011_{AB}^{e}$	$0.463\pm 0.01_{A}$	0.000
T2	$0.405\pm 0.007^{a}$	$0.428\pm 0.008_{B}^{\mathrm{ab}}$	$0.458\pm 0.009^{bc}$	$0.489\pm 0.012_{A}^{cd}$	$0.519\pm 0.012_{AB}^{d}$	$0.561\pm 0.015_{\mathrm{AB}}^{e}$	$0.477\pm 0.01_{AB}$	0.000
T3	$0.407\pm 0.006^{a}$	$0.435\pm 0.01_{B}^{\mathrm{ab}}$	$0.470\pm 0.02^{b}$	$0.533\pm 0.013_{B}^{c}$	$0.548\pm 0.011_{B}^{cd}$	$0.578\pm 0.011_{B}^{d}$	$0.495\pm 0.01_{B}$	0.00
Storage mean	$0.397\pm 0.004^{c}$	$0.419\pm 0.005^{b}$	$0.449\pm 0.006^{b}$	$0.491\pm 0.008_{A}^{a}$	$0.518\pm 0.006_{A}^{a}$	$0.553\pm 0.007^{c}$	−	0.00
*p*-value	0.063	0.035	0.072	0.005	0.018	0.05	0.017	−
**Water activity (a_W_)**								
C	$0.930\pm 0.009^{d}$	$0.913\pm 0.007_{A}^{cd}$	$0.896\pm 0.007^{c}$	$0.874\pm 0.007_{A}^{b}$	$0.863\pm 0.008_{A}^{ab}$	$0.848\pm 0.008_{A}^{a}$	$0.887\pm 0.006_{A}$	0.000
T1	$0.932\pm 0.008^{d}$	$0.927\pm 0.002_{AB}^{cd}$	$0.911\pm 0.006^{bc}$	$0.904\pm 0.005_{B}^{\mathrm{ab}}$	$0.894\pm 0.007_{B}^{\mathrm{ab}}$	$0.887\pm 0.007_{B}^{a}$	$0.909\pm 0.004_{B}$	0.000
T2	$0.934\pm 0.006^{c}$	$0.921\pm 0.006_{A}^{bc}$	$0.917\pm 0.007^{bc}$	$0.908\pm 0.006_{B}^{b}$	$0.902\pm 0.006_{B}^{b}$	$0.881\pm 0.01_{B}^{a}$	$0.911\pm 0.003_{B}$	0.000
T3	$0.935\pm 0.02^{cd}$	$0.936\pm 0.004_{B}^{d}$	$0.925\pm 0.01^{bcd}$	$0.914\pm 0.003_{B}^{\mathrm{bc}}$	$0.908\pm 0.003_{B}^{\mathrm{ab}}$	$0.889\pm 0.008_{B}^{a}$	$0.918\pm 0.003_{B}$	0.00
Storage means	$0.928\pm 0.004^{c}$	$0.925\pm 0.003^{b}$	$0.913\pm 0.004^{b}$	$0.900\pm 0.004^{a}$	$0.892\pm 0.005^{a}$	$0.876\pm 0.005^{a}$	−	0.00
*p*-value	0.996	0.029	0.064	0.000	0.000	0.008	0.000	−

Means bearing different superscripts with small letters a, b, c, d, e and subscripts with capital letters A, B along a row and column, respectively, differ significantly at *p* ≤ 0.05. C: Control paneer without turmeric and aloe vera extract, T1: Paneer with turmeric extract (5 mL/100 mL) *v*/*v* of milk; T2: Paneer with aloe vera extract (5 mL/100 mL) *v*/*v* of milk; T3: paneer with turmeric extract (5 mL/100 mL) *v*/*v* of milk and aloe vera extract (5 mL/100 mL) *v*/*v* of milk.

**Table 3:** Effect of turmeric and aloe vera extracts on the microbial quality of goat and buffalo paneer during refrigerated aerobic storage.

Groups	0 Day	2 Days	4 Days	6 Days	8 Days	10 Days	Group Means	*p*-Value
**Standard Plate Counts (cfu/g)**								
C	$2.73\pm 0.08^{a}$	$3.00\pm 0.05^{b}$	$3.51\pm 0.11^{c}$	$4.06\pm 0.13_{B}^{d}$	$5.17\pm 0.08_{C}^{e}$	$6.11\pm 0.06_{C}^{f}$	4.10±0.21	0.000
T1	$2.63\pm 0.06^{a}$	$2.87\pm 0.08^{ab}$	$3.12\pm 0.14^{b}$	$3.63\pm 0.06_{A}^{c}$	$4.60\pm 0.09_{AB}^{d}$	$5.48\pm 0.08_{B}^{e}$	3.72±0.17	0.000
T2	$2.69\pm 0.07^{a}$	$2.89\pm 0.10^{a}$	$3.58\pm 0.10^{b}$	$3.73\pm 0.08_{A}^{c}$	$4.74\pm 0.08_{B}^{d}$	$5.87\pm 0.07_{C}^{e}$	3.88±0.19	0.000
T3	$2.61\pm 0.06^{a}$	$2.80\pm 0.07^{a}$	$3.44\pm 0.08^{b}$	$3.62\pm 0.10_{A}^{b}$	$4.43\pm 0.10_{A}^{c}$	$5.22\pm 0.12_{A}^{d}$	3.66±0.16	0.00
Storage mean	$2.67\pm 0.03^{a}$	$2.89\pm 0.04^{b}$	$3.36\pm 0.06^{c}$	$3.71\pm 0.06^{d}$	$4.74\pm 0.07^{e}$	$5.67\pm 0.08^{f}$	−	0.00
*p*-value	0.000	0.350	0.105	0.001	0.000	0.000	0.329	−
**Psychrophilic counts (cfu/g)**								
C	$1.88\pm 0.12^{a}$	$2.33\pm 0.09^{b}$	$2.77\pm 0.15_{C}^{c}$	$2.99\pm 0.18_{B}^{c}$	$3.61\pm 0.09_{B}^{d}$	$3.85\pm 0.08_{C}^{d}$	$2.91\pm 0.13_{C}$	0.000
T1	$1.57\pm 0.19^{a}$	$1.99\pm 0.08^{b}$	$2.30\pm 0.06_{B}^{\mathrm{bc}}$	$2.57\pm 0.07_{\mathrm{AB}}^{\mathrm{cd}}$	$2.91\pm 0.14_{A}^{d}$	3.05$\pm 0.12_{AB}^{e}$	$2.40\pm 0.10_{AB}$	0.000
T2	$1.65\pm 0.14^{a}$	$2.12\pm 0.12^{b}$	$2.46\pm 0.09_{BC}^{bc}$	$2.72\pm 0.11_{AB}^{c}$	$2.82\pm 0.08_{A}^{\mathrm{cd}}$	$3.16\pm 0.17_{B}^{d}$	$2.49\pm 0.09_{B}$	0.000
T3	$1.52\pm 0.13^{a}$	$1.87\pm 0.20^{a}$	$1.84\pm 0.12_{A}^{a}$	$2.33\pm 0.13_{A}^{b}$	$2.58\pm 0.15_{A}^{b}$	$2.76\pm 0.06_{A}^{b}$	$2.15\pm 0.09_{A}$	0.00
Storage means	$1.66\pm 0.08^{a}$	$2.08\pm 0.07^{b}$	$2.34\pm 0.09^{c}$	$2.66\pm 0.08^{d}$	$2.98\pm 0.10^{e}$	$3.21\pm 0.10^{e}$	−	0.00
*p*-value	0.364	0.110	0.000	0.021	0.000	0.000	0.000	−
**Coliform counts (cfu/g)**								
C	ND	ND	ND	$1.32\pm 0.11^{a}$	$1.56\pm 0.11_{A}^{a}$	$2.40\pm 0.15_{C}^{b}$	$0.887\pm 0.006_{A}$	0.000
T1	ND	ND	ND	ND	$1.29\pm 0.11_{B}^{a}$	$1.35\pm 0.10_{A}^{a}$	$0.909\pm 0.004_{B}$	0.000
T2	ND	ND	ND	ND	$1.41\pm 0.10_{B}^{a}$	$1.84\pm 0.10_{B}^{b}$	$0.911\pm 0.003_{B}$	0.000
T3	ND	ND	ND	ND	ND	$1.27\pm 0.06_{A}^{a}$	$0.918\pm 0.003_{B}$	0.00
Storage means	−	−	−	$0.33\pm 0.12^{a}$	$1.06\pm 0.13^{b}$	$1.72\pm 0.11^{c}$	−	0.00
*p*-value	−	−	−	0.000	0.000	0.000	0.003	−
**Yeast and mould counts (cfu/g)**								
C	ND	ND	ND	$1.86\pm 0.13_{A}^{a}$	$2.12\pm 0.07_{B}^{b}$	$2.29\pm 0.11_{B}^{b}$	$0.887\pm 0.006_{A}$	0.000
T1	ND	ND	ND	ND	$1.85\pm 0.10_{A}^{a}$	$2.18\pm 0.10_{B}^{b}$	$0.909\pm 0.004_{B}$	0.000
T2	ND	ND	ND	$1.67\pm 0.15_{A}^{a}$	$1.93\pm 0.11_{\mathrm{AB}}^{b}$	$2.03\pm 0.08_{\mathrm{AB}}^{b}$	$0.911\pm 0.003_{B}$	0.000
T3	ND	ND	ND	ND	$1.75\pm 0.04_{A}^{a}$	$1.82\pm 0.07_{A}^{a}$	$0.918\pm 0.003_{B}$	0.00
Storage means	−		−	$0.88\pm 0.19^{a}$	$1.92\pm 0.05^{b}$	$2.08\pm 0.06^{b}$	−	0.00
*p*-value	−	−	−	0.000	0.034	0.010	0.000	−

Means bearing different superscripts with small letters a, b, c, d, e, f and subscripts with capital letters A, B, C along a row and column respectively differ significantly at *p* ≤ 0.05. C: Control paneer without turmeric and aloe vera extract, T1: Paneer with turmeric extract (5 mL/100 mL) *v*/*v* of milk; T2: Paneer with aloe vera extract (5 mL/100 mL) *v*/*v* of milk; T3: paneer with turmeric extract (5 mL/100 mL) *v*/*v* of milk and aloe vera extract (5 mL/100 mL) *v*/*v* of milk.

**Table 4:** Effects of turmeric and aloe vera extracts on sensory attributes of goat and buffalo paneer during refrigerated aerobic storage.

Groups	0 Day	2 Days	4 Days	6 Days	8 Days	10 Days	Group Means	*p*-Value
**Colour & Appearance**								
C	$8.14\;\pm \;0.09^{e}$	$7.89\pm 0.09^{e}$	$6.96\pm 0.12_{A}^{d}$	$6.64\pm 0.09_{A}^{c}$	$6.14\pm 0.07_{A}^{b}$	$5.57\pm 0.12_{A}^{a}$	$6.89\pm 0.15_{A}$	0.000
T1	$7.93\pm 0.09^{e}$	$7.64\pm 0.07^{\mathrm{de}}$	$7.36\pm 0.11_{B}^{d}$	$7.00\pm 0.10_{B}^{c}$	$6.57\pm 0.11_{B}^{b}$	$6.25\pm 0.11_{B}^{a}$	$7.13\pm 0.10_{AB}$	0.000
T2	$8.04\pm 0.10^{d}$	$7.86\pm 0.09^{e}$	$7.54\pm 0.10_{BC}^{d}$	$7.07\pm 0.07_{B}^{c}$	$6.32\pm 0.11_{\mathrm{AB}}^{b}$	$5.79\pm 0.14_{A}^{a}$	$7.10\pm 0.13_{AB}$	0.000
T3	$8.00\pm 0.11^{c}$	$7.82\pm 0.11^{c}$	$7.71\pm 0.13_{C}^{c}$	$7.18\pm 0.07_{B}^{b}$	$7.00\pm 0.08_{C}^{\mathrm{ab}}$	$6.71\pm 0.15_{C}^{a}$	$7.41\pm 0.08_{B}$	0.00
Storage mean	$8.03\pm 0.05^{f}$	$7.80\pm 0.05^{e}$	$7.39\pm 0.08^{d}$	$6.97\pm 0.06^{c}$	$6.51\pm 0.08^{b}$	$6.08\pm 0.11^{a}$	−	0.00
*p*-value	0.493	0.248	0.001	0.001	0.001	0.000	0.027	−
**Flavour**								
C	$8.21\pm 0.08^{f}$	$7.79\pm 0.07^{e}$	$7.21\pm 0.13^{d}$	$6.29\pm 0.10_{A}^{c}$	$6.00\pm 0.08_{A}^{b}$	$5.64\pm 0.11_{A}^{a}$	$6.86\pm 0.15_{A}$	0.000
T1	$8.04\pm 0.07^{e}$	$7.68\pm 0.07^{d}$	$7.32\pm 0.11^{c}$	$7.18\pm 0.07_{B}^{c}$	$6.75\pm 0.11_{BC}^{b}$	$6.46\pm 0.13_{BC}^{a}$	$7.24\pm 0.09_{B}$	0.000
T2	$8.11\pm 0.12^{f}$	$7.75\pm 0.08^{e}$	$7.43\pm 0.07^{d}$	$7.11\pm 0.09_{B}^{c}$	$6.64\pm 0.13_{B}^{b}$	$6.17\pm 0.15_{B}^{a}$	$7.20\pm 0.11_{B}$	0.000
T3	$8.00\pm 0.12^{d}$	$7.79\pm 0.14^{cd}$	$7.50\pm 0.08^{bc}$	$7.32\pm 0.11_{B}^{b}$	$6.96\pm 0.07_{C}^{a}$	$6.75\pm 0.11_{C}^{a}$	$7.39\pm 0.08_{B}$	0.00
Storage mean	$8.09\pm 0.05^{f}$	$7.75\pm 0.05^{e}$	$7.37\pm 0.05^{d}$	$6.97\pm 0.09^{c}$	$6.59\pm 0.08^{b}$	$6.26\pm 0.10^{a}$	−	0.00
*p*-value	0.4878	0.828	0.208	0.000	0.000	0.000	0.008	−
**Texture**								
C	$8.18\pm 0.07^{f}$	$7.71\pm 0.09_{\mathrm{AB}}^{e}$	$7.14\pm 0.07_{A}^{d}$	$6.61\pm 0.008_{A}^{c}$	$6.11\pm 0.09_{A}^{b}$	$5.39\pm 0.09_{A}^{a}$	$6.80\pm 0.11_{A}$	0.000
T1	$8.07\pm 0.07^{d}$	$7.54\pm 0.14_{A}^{c}$	$7.29\pm 0.12_{A}^{bc}$	$7.04\pm 0.07_{B}^{b}$	$6.57\pm 0.12_{B}^{a}$	$6.29\pm 0.08_{B}^{a}$	$7.13\pm 0.10_{AB}$	0.000
T2	$8.11\pm 0.09^{d}$	$7.89\pm 0.09_{B}^{d}$	$7.32\pm 0.11_{A}^{c}$	$7.21\pm 0.09_{\mathrm{BC}}^{c}$	$6.79\pm 0.09_{B}^{b}$	$6.39\pm 0.09_{B}^{a}$	$7.29\pm 0.09_{B}$	0.000
T3	$8.21\pm 0.09^{e}$	$8.00\pm 0.10_{B}^{e}$	$7.68\pm 0.13_{B}^{d}$	$7.36\pm 0.36_{C}^{c}$	$6.86\pm 0.09_{B}^{b}$	$6.46\pm 0.13_{B}^{a}$	$7.43\pm 0.10_{B}$	0.00
Storage means	$8.14\pm 0.04^{f}$	$7.79\pm 0.06^{e}$	$7.36\pm 0.07^{d}$	$7.05\pm 0.07^{c}$	$6.58\pm 0.07^{b}$	$6.13\pm 0.10^{a}$	−	0.00
*p*-value	0.587	0.023	0.013	0.000	0.000	0.000	0.005	−
**Overall acceptability**								
C	$8.14\pm 0.07^{f}$	$7.79\pm 0.09^{e}$	$7.54\pm 0.07^{d}$	$6.82\pm 0.11_{A}^{c}$	$6.21\pm 0.09_{A}^{b}$	$5.51\pm 0.08_{A}^{a}$	$7.00\pm 0.15_{A}$	0.000
T1	$8.07\pm 0.09^{e}$	$7.64\pm 0.11^{d}$	$7.39\pm 0.09^{d}$	$7.11\pm 0.05_{B}^{c}$	$6.79\pm 0.09_{B}^{b}$	$6.43\pm 0.11_{B}^{a}$	$7.24\pm 0.09_{AB}$	0.000
T2	$8.11\pm 0.09^{f}$	$7.71\pm 0.09^{e}$	$7.43\pm 0.12^{d}$	$6.96\pm 0.07_{\mathrm{AB}}^{c}$	$6.54\pm 0.10_{B}^{b}$	$6.25\pm 0.12_{B}^{a}$	$7.17\pm 0.11_{A}$	0.000
T3	$8.18\pm 0.07^{e}$	$7.93\pm 0.07^{d}$	$7.71\pm 0.09^{c}$	$7.39\pm 0.09_{C}^{b}$	$7.25\pm 0.08_{C}^{b}$	$6.75\pm 0.11_{C}^{a}$	$7.54\pm 0.08_{B}$	0.00
Storage means	$8.13\pm 0.04^{f}$	$7.77\pm 0.05^{e}$	$7.52\pm 0.05^{d}$	$7.07\pm 0.06^{c}$	$6.70\pm 0.08^{b}$	$6.23\pm 0.10^{a}$	−	0.00
*p*-value	0.816	0.154	0.088	0.000	0.000	0.000	0.007	−

Means bearing different superscripts with small letters a, b, c, d, e, f and subscripts with capital letters A, B, C along a row and column respectively differ significantly at *p* ≤ 0.05. C: Control paneer without turmeric and aloe vera extracts, T1: Paneer with turmeric extract (5 mL/100 mL) *v*/*v* of milk; T2: Paneer with aloe vera extract (5 mL/100 mL) *v*/*v* of milk; T3: paneer with turmeric extract (5 mL/100 mL) *v*/*v* of milk and aloe vera extract (5 mL/100 mL) *v*/*v* of milk.

## Data Availability

Data is contained within the article.
